# Supplementary material for: Health system capacity and readiness for delivery of integrated non-communicable disease services in primary health care: A qualitative analysis of the Ethiopian experience
Source: PLOS Glob Public Health. 2021 Oct 13;1(10):e0000026. doi: 10.1371/journal.pgph.0000026 (PMC10021149; doi:10.1371/journal.pgph.0000026)
Supplement: S1 File — (DOCX) [file pgph.0000026.s001.docx]

**I. Participant information statement and consent form**

1. **What is the research study about?**

The research study aims to explore the system approach to understanding and improving the delivery of Primary Health Care in Ethiopia. You have been invited because you are part of the policy makers/program coordinator/primary health care worker in Ethiopia and your contact was obtained from the Ministry of Health/regional health bureau/woreda(district) health office/your health facility.

1. **Who is conducting this research?**

The study is being carried out by *The George Institute for Global Health, Faculty of Medicine, University of New South Wales, Sydney staff members.*

**Research Funder:** This research is being funded by The George Institute for Global Health, Australia with a seed grant to support research for Under-served community in low-middle Income countries.

1. **Inclusion/Exclusion Criteria**

Before you decide to participate in this research study, we need to ensure that it is ok for you to take part. The research study is looking to recruit people who meet any of the following criteria:

- You are one of the policy makers on health related by the virtue of your position
- You are currently in one of this categories; representatives from the Health extension and Primary Health Service Directorate (Federal MOH)/ national and regional HEP coordinators/ regional Health promotion and diseases prevention team leaders/ programme manager/ HEP supervisor/health care worker

1. **Do I have to take part in this research study?**

Participation in this research study is voluntary. If you do not want to take part, you do not have to. If you decide to take part and later change your mind, you are free to withdraw from the study at any stage.

If you decide you want to take part in the research study, you will be asked to:

- Read the information carefully (ask questions if necessary);
- Sign and return the consent form if you decide to participate in the study;
- Take a copy of this form with you to keep.

1. **What does participation in this research require, and are there any risks involved?**

Participation in an interview

- If you decide to take part in the research study, you will be asked to participate in a face to face interview. You will be asked questions about the overall process and the challenges faced by policy makers in providing health care services for non-communicable diseases at the level of the community. It should take approximately **45-60 minutes** to complete.
- To ensure we collect the responses accurately, we seek your permission to digitally record the interview using an audio tape. If you would like to participate but do not wish to be recorded, you will need to discuss the options for your participation with the research team. For this, the research team we will ask you to sign the consent form and then will continue with the questions. This process will take approximately **10-15 minutes** to complete.

1. **What are the possible benefits to participation?**

We hope to use information we get from this research study to benefit others who seek to understand a system approach to delivering NCD services through non-physician health workers at the primary health care level. This can also help in addressing challenges faced by the health workers and the system at this level.

1. **What will happen to information about me?**

By signing the consent form, you consent to the research team collecting and using information about you for the research study. Your data will be kept for a minimum of 5 years after the publication of the outcome of this research or after the project’s completion. We will store information about you in a non-identifiable format at the George Institute for Global Health office in Sydney, Australia. Your information will only be used for this research study only. Although the results of this research study will be published and/or presented in a variety of forums but in any publication and/or presentation, information will be published in a way such that you will not be individually identifiable.

The information you provide is personal information for the purposes of the Privacy and Personal Information Protection Act 1998 (NSW). You have the right of access to personal information held about you by the University, the right to request correction and amendment of it, and the right to make a complaint about a breach of the Information Protection Principles as contained in the PPIP Act. Further information on how the University protects personal information is available in the [**UNSW Privacy Management Plan**](https://www.legal.unsw.edu.au/compliance/privacyhome.html).

1. **How and when will I find out what the results of the research study are?**

The research team intend to publish and/ report the results of the research study in a variety of ways. All information published will be done in a way that will not identify you.

If you would like to receive a copy of the results you can let the research team know by including your details in the space provided in the consent form.

1. **What if I want to withdraw from the research study?**

If you do consent to participate, you may withdraw at any time. You can do so by completing the ‘Withdrawal of Consent Form’ which is provided at the end of this document. Alternatively, you can ring the research team particularly Azeb Tesema and tell them you no longer want to participate. Your decision not to participate or to withdraw from the study will not affect your relationship with UNSW Sydney or The George Institute for Global Health, Australia.

If you decide to leave the research study, the researchers will not collect additional information from you. Any identifiable information about you will be withdrawn from the research project.

1. **What should I do if I have further questions about my involvement in the research study?**

The person you may need to contact will depend on the nature of your query. If you require further information regarding this study or if you have any problems which may be related to your involvement in the study, you can contact the following member/s of the research team:

**Research Team Contact Details**

| **Name** | Azeb G. Tesema |
| --- | --- |
| **Position** | Student investigator |
| **Email** | a.tesema@student.unsw.edu.au |

**What if I have a complaint or any concerns about the research study?**

If you have a complaint regarding any aspect of the study or the way it is being conducted, please contact the UNSW Human Ethics Coordinator: [humanethics@unsw.edu.au](mailto:humanethics@unsw.edu.au)

**Consent Form – Participant providing own consent**

**Declaration by the participant**

- I understand I am being asked to provide consent to participate in this research study;
- I have read the Participant Information Sheet or someone has read it to me in a language that I understand;
- I understand the purposes, study tasks and risks of the research described in the study;
- I understand that the research team will audio record the interviews; I agree to be recorded for this purpose.
- I provide my consent for the information collected about me to be used for the purpose of this research study only.
- I have had an opportunity to ask questions and I am satisfied with the answers I have received;
- I freely agree to participate in this research study as described and understand that I am free to withdraw at any time during the study and withdrawal will not affect my relationship with any of the named organisations and/or research team members;
- I would like to receive a copy of the study results via email or post, I have provided my details below and ask that they be used for this purpose only;

**Name: _____________________________________**

**Address: ___________________________________**

**Email Address: ______________________________**

- I understand that I will be given a signed copy of this document to keep;

**Participant Signature**

| Name of Participant (please print) |  |
| --- | --- |
| Signature of Research Participant |  |
| Date |  |

**Declaration by Researcher***

- I have given a verbal explanation of the research study, its study activities and risks and I believe that the participant has understood that explanation.

**Researcher Signature***

| Name of Researcher (please print) |  |
| --- | --- |
| Signature of Researcher |  |
| Date |  |

**^+^An appropriately qualified member of the research team must provide the explanation of, and information concerning the research study.**

**Note: All parties signing the consent section must date their own signature.**

**Form for Withdrawal of Participation**

I wish to **WITHDRAW** my consent to participate in this research study described above and understand that such withdrawal **WILL NOT** affect my relationship with The University of New South Wales and The George Institute for Global Health, Australia. In withdrawing my consent I would like any information which I have provided for the purpose of this research study withdrawn.

**Participant Signature**

| Name of Participant  (please print) |  |
| --- | --- |
| Signature of Research Participant |  |
| Date |  |

**The section for Withdrawal of Participation should be forwarded to:**

| CI Name: | David Peiris |
| --- | --- |
| Email: | [dpeiris@georgeinstitute.org.au](mailto:dpeiris@georgeinstitute.org.au) |

**II. Interview guides**

**A. Key informant interview guide for policy makers (National, regional, woreda(district) health office representatives, and program coordinators)**

**The aim of the Interview**

This interview is part of the research study “exploring the role of the primary health care in NCDs prevention and management program in Ethiopia, focusing on health extension program”. This study is conducted by George Institute for Global Health Australia and the University of New South Wales (UNSW), Sydney.

| **S.No** | **Main question/exploratory questions** | **Probing questions/ Tell me more about** |
| --- | --- | --- |
| 1 | **Introductory question**  Tell me your experience/opinion about the Ethiopian primary health care (PHC) and health extension program (HEP), in terms of the overall health system structure (federal or policy level, at the lower level) |  |
| 2 | What kind of policy, strategy and guideline exist in Ethiopian health system to prevent, manage and control NCDs. Especially at the PHC level | What actions are implemented at the national level for prevention of NCDs, and what about the at the health care level  What is the plan to address the NCDs program at the community level? |
| 3 | What is the role of PHC in terms of NCDs prevention and management (eg. CVD and diabetes prevention and control)? |  |
| 4 | In your opinion, how do you evaluate the readiness of the PHC unit especially the HEP in delivering NCD prevention and control program? | Do the region/woreda realise the burden of NCDs (diabetic and hypertension) in the population |
| 5 | Would you please explain the inter sectoral collaboration and even inter departmental relationship in addressing the burden of NCDs | Eg. for the implementation of NCDs at the PHC level, how do you evaluate the harmonization of the activities among your team and the HEP team |
| 6 | In your opinion, how do you see the attention given for NCD program different levels?  Governance: in your observation, do you think the government/high level officials are giving attention for NCDs? | Focus person delegated for NCDs at the regional level/woreda level  Is the PHC board members aware about NCDs? |
| 7 | Is there clear allocated budget for NCDs program (specially at the PHCs level) | What kind of financial mechanism can be proposed for primary health care for NCDs |
| 8 | Please tell me about national/region/woreda commitment to equip the primary health care unit in terms of equipment and supply | What protocols, guidelines, and other teaching aid are available for the NCD program?  What is your view in using technology to assist the implementation of the program (e.g m-health) |
| 9 | Would you please tell us about the supportive supervision process that exist in the PHC system? |  |
| 10 | What are the strategies designed to engage the community in NCDs prevention and control program |  |
| 11 | What policy direction implemented at the ground to improve information management systems at the PHC unit/especially at health post level? | Does the existing system include the NCDs data? If not why? |
| 12 | Referral System:  Would you please tell me about the referral system? | What are the challenge for good referral, especially for NCDs care?   - What policy direction are proposed to improve the referral system? |
| 13 | What are the biggest challenges for addressing NCDs through the PHC/HEP in the health system? | What do you recommend? |

**B. In-depth interview guide for Primary hospital and health centre participants**

| **S.No** | **Main question/exploratory questions** | **Probing questions** |
| --- | --- | --- |
| **1** | Please tell me your experience/opinion about the PHC/HEP in terms of the overall health system structure |  |
| 2 | Please tell me the role of the PHC unit for NCD prevention and management (like cardiovascular disease and diabetes)?  What about the role of HEWs in NCDs prevention and management program (CVD and diabetes prevention and control) | - Is there any challenges in implementing NCD program at the PHC level? |
| 3 | How do you evaluate the readiness of your health facility and the cluster health posts to implement NCD prevention and control program?  What should be done to improve its implementation? | What can be done/ planning to do to integrate the NCDs service at the health centre and health post level |
| 4 | How do you see the role of the governing boards or PHC board members in NCDs activities?  how do you ensure coordination of the NCD program in your facility? | What about accountability of the leaders for implementation NCDs? |
| 5 | Is there any trainings on NCD given for PHCU staffs including the HEWs? | Quality, adequacy  Ways to integrate the NCDs training program the existing Integrated refreshment training program |
| 6 | How do you supervise the HEWs in your cluster?  What are the challenges with the supervision process?  Specially to implement the NCDs service? | Do you think the supervision is supportive and improve the knowledge and skill of the HEWs? |
| 7 | Please tell me about the provision of equipment and supplies for the PHCU to provide NCD services? |  |
| 8 | How do you see the support of the community to implement of NCD prevention and management? |  |
| 9 | What strategy is implemented to integrate the NCDs data management information system at PHC unit level?  What support are you providing for your cluster health post to improve the information management system? |  |
| 10 | How do you see the referral system in the PHCU in relation to NCDs case management? | What are the challenge for good referral, especially for NCDs care? |

**C. In-depth interview guide for Health extension workers (HEWs)**

| **S.No** | **Main question/exploratory questions** | **Probing questions** |
| --- | --- | --- |
| 1 | Please tell me your experience as Health extension worker (HEW) |  |
| 2 | What specifically are you doing for non-communicable disease prevention and management (especially for cardio-vascular diseases and Diabetes)? | Which area of work are you focusing for non-communicable diseases (health education on prevention of risk factors, screening or other?) |
|  | -Do you think the type of supervision you are getting now is helpful especially for you to get the required knowledge and skill in NCD service provision? | Is there any challenges relating the supportive supervision with your overall performance? |
| 4 | **Incentives**  Can you tell us your experience, related to what motivate/demotivate you to work as health extension works?  What incentives do you think would motivate Health extension workers to retain their job? |  |
| 5 | **Social issues**  What is your view about your additional role in the implementation of NCD program? | Do you think it is a burden (work load?)  What kind of improvement you want to suggest managing the challenge? (increase the number of health extension workers? |
| 6 | What can be done to engage the community in NCD prevention and management |  |
| 7 | Referral System  What is the process to refer patients for health services to the higher level?  What about for non-communicable diseases? |  |
| 8 | What is your readiness to implement NCD prevention and management? | What do you think will be the biggest challenge? |
