# Supplementary material for: Health system capacity and readiness for delivery of integrated non-communicable disease services in primary health care: A qualitative analysis of the Ethiopian experience
Source: PLOS Glob Public Health. 2021 Oct 13;1(10):e0000026. doi: 10.1371/journal.pgph.0000026 (PMC10021149; doi:10.1371/journal.pgph.0000026)
Supplement: Attachment — Submitted filename: Response to Reviewers.docx [file pgph.0000026.s002.docx]

11^th^ August 2021

**Author Response Letter: PLOS Global Public Health [PGPH-D-21-00231]**

Dear Dr. Muhammad Fawad Rasool/Academic Editor

Thank you for reviewing our manuscript on “*Health system capacity and readiness for delivery of integrated Non-communicable disease services in Primary Health Care: A qualitative analysis of the Ethiopian experience*”. We have revised the manuscript to respond to the points raised by you and the reviewers and uploaded a clean and tracked change manuscript.

**Journal requirements**

- *Please review your reference list to ensure that it is complete and correct. If you have cited papers that have been retracted, please include the rationale for doing so in the manuscript text or remove these references and replace them with relevant current references. Any changes to the reference list should be mentioned in the rebuttal letter that accompanies your revised manuscript. If you need to cite a retracted article, indicate the article’s retracted status in the References list and also include a citation and full reference for the retraction notice.*

We have not used retracted papers; however, there were citation errors for references four and five. The reference list is now up to date.

- *While revising your submission, please upload your figure files to the Preflight Analysis and Conversion Engine (PACE) digital diagnostic tool, https://pacev2.apexcovantage.com/. PACE helps ensure that figures meet PLOS requirements.*

We uploaded the figures into the Preflight Analysis and Conversion Engine (PACE) digital diagnostic tool and checked their quality. Now, the figures meet the PLOS requirements.

**Reviewers' comments:**

**Reviewer 1:**

I. *There are only two notes for minor revision:*

*A- In the abstract method, the sentence “in two regions and Addis Ababa, Ethiopia” after two regions, the “and” does not make sense.*

We have clarified the sentence and in the revised manuscript, it read as “in two regions and Federal Ministry of Health, Addis Ababa, Ethiopia”. Please also see the abstract section of the manuscript.

*B- In the main method body, study settings section, please clarify “woredas” as it is not an English word.*

We have clarified the idea in the revised manuscript, as woreda is equivalent to the district. Please see the study setting part of the method section on page 5.

**Reviewer 2:**

I. *To me although there is some needs to know more about the Ethiopian heath numerical indicators to understand the essentiality of the study and the burden of NCD in the country, but the research well designed and has value to study. Also, I expect to get the answers for three main objectives more typically in the conclusion.*

We believe that our paper addresses the objectives in an interrelated and detailed way. We have summarised the results of the 3 aims (1. strategic and operational levers needed to facilitate the implementation of NCDs services in PHC 2. how can the health system build on the past success stories and lessons to integrate NCD prevention and care? And 3. what additional enabling elements already exist in the system that can be harnessed and capitalised on for optimal NCD service integration into PHC service delivery models?) in the conclusions and in figure 2. Page 19, paragraphs 2 and 3.

Thank you again for your feedback and your time to review our paper. Please contact me if you have any further questions.

Best regards,

Azeb Gebresilassie Tesema

ORCID iD: https://orcid.org/0000-0003-0618-4499
